# Percutaneous Tibial Nerve Stimulation: A Clinically and Cost Effective Addition to the Overactive Bladder Algorithm of Care

**DOI:** 10.1007/s11934-012-0274-9

**Published:** 2012-08-15

**Authors:** David R. Staskin, Kenneth M. Peters, Scott MacDiarmid, Neal Shore, William C. de Groat

**Affiliations:** 1 Department of Urology, Tufts University School of Medicine, Boston, MA USA; 2 Department of Urology, William Beaumont Hospital, Royal Oak, MI USA; 3 Alliance Urology Specialists, Greensboro, NC USA; 4 Department of Urology, Carolina Urologic Research Center, Myrtle Beach, SC USA; 5 Department of Pharmacology, University of Pittsburgh School of Medicine, Pittsburgh, PA USA; 6 Division of Urology, Steward Health - St Elizabeth’s Medical Center, 11 Nevins St. DOB-303, Boston, MA 02135 USA

**Keywords:** Electric stimulation therapy, Urinary bladder, Overactive, Cost-benefit, Electrodes, Stimulation, Overactive detrusor activity

## Abstract

Overactive bladder affects millions of adults, with profound personal and economic costs. Although antimuscarinic drugs can cause a reduction in voiding symptoms, the effect is modest, and many patients are intolerant of the side effects, or do not experience sufficient relief. For these patients, the modulation of bladder reflex pathways via percutaneous tibial nerve stimulation (PTNS) or via implanted sacral nerve stimulation (SNS) has been acknowledged as a logical next step in the algorithm of care. This review examines the mechanism of action, the relative benefits, adverse effects, and costs of percutaneous nerve stimulation compared to other treatment modalities.

## Introduction

When behavioral therapy or pharmacology is not effective in the treatment of overactive bladder (OAB), the modulation of bladder reflex pathways has been acknowledged as the next logical step in the algorithm of care. This care path has recently been codified in the 2012 American Urological Association guidelines for the diagnosis and treatment of OAB [1••]. The two most commonly utilized neuromodulation techniques are percutaneous tibial nerve stimulation (PTNS) and sacral nerve stimulation (SNS). Of note, although both PTNS and SNS are believed to modulate neural pathways, PTNS and SNS are believed to target different neural circuitry in the central nervous system. In addition, PTNS uses intermittent (weekly) stimulation of the tibial nerve at the ankle with no permanent lead or stimulator implanted, while SNS provides continuous stimulation through surgical implantation of a permanent electrode and a permanent pulse generator.

Based on three randomized trials and one long-term durability trial [2–6, 7••], the National Institute for Health and Clinical Excellence (NICE) in the United Kingdom, issued guidance in October 2010 stating, “PTNS for OAB demonstrates effectiveness without major safety concerns” [8]. The United States Food and Drug Administration (FDA) has cleared PTNS for treatment of overactive bladder and the associated symptoms of urinary frequency, urinary urgency and urinary urge incontinence, and a category I CPT code was approved by the Centers for Medicare and Medicaid Services (effective Jan. 1, 2011).

This review presents the currently available data regarding the efficacy, long-term durability, adverse treatment effects, and costs of percutaneous tibial nerve stimulation in comparison with other OAB treatment modalities. This review will discuss the place of PTNS in the current and evolving OAB treatment algorithm, particularly for those patients in whom pharmacological therapy does not meet their expectations (lack of efficacy or poor tolerability). Behavioral therapy is not included in this review as it is acknowledged as a stand-alone treatment that can be used in combination with other OAB therapies including pharmacotherapy or neuromodulation. Botulinum toxin is currently not FDA approved for idiopathic OAB, and is not included in this review. Similarly, although augmentation cystoplasty has been employed as a surgical alternative, the utilization of this relatively invasive intra-abdominal procedure is limited.

## Background

### Overactive Bladder (OAB)

Approximately 34 million adults in the United States suffer daily from Overactive Bladder (OAB) [9], defined by The International Continence Society (ICS) as a syndrome with or without urgency incontinence, usually associated with urinary frequency and nocturia in the absence of proven infection or other obvious pathology [10]. OAB affects men and women equally, with women more likely to have urinary urge incontinence than men (9.3 % vs. 2.6 %) and with increasing incidence with age [9]. Urinary incontinence can result in social isolation and depression, employment disruption, as well as adversely impacting a patient’s quality of life and sexual functioning [11]. Overall health care costs in the USA attributed to OAB, including lost productivity, have been estimated to be more than $65 billion per year [12]. Urinary incontinence has also been linked to increased morbidity, causing additional traumatic falls with associated fractures [13]. Literature estimates for annual incidence of OAB range from 2.6–143 cases per thousand, equivalent to a 12 % prevalence rate, with the majority of patients suffering with symptoms for years [14]. Although there are a wide variety of OAB treatment options, improvement in symptoms is ultimately the primary goal, as complete eradication and resolution of chronic OAB symptoms is rarely achieved.

The sine qua non of urgency incontinence or OAB-wet is overactive detrusor activity during bladder filling, although symptoms of urgency may occur in the absence of detrusor activity [11]. The first-line treatments for OAB are behavioral therapies aimed at altering the patient’s habits and response to the subjective sense of urgency [1••]. Although these therapies are often effective, many patients do not improve enough to meet their expectations. In these non-responders, pharmacological therapy focuses on suppression of bladder symptoms by blocking cholinergic-muscarinic receptors in the bladder with antimuscarinic drugs. When behavioral therapy or pharmacologic strategies are not effective, the modulation of bladder reflex pathways with neuromodulation has been suggested as the next therapeutic intervention. The utilization of intravesical botulinum toxin injection for refractory idiopathic OAB patients is awaiting FDA approval and clinical integration and is not discussed in this review.

### Percutaneous Tibial Nerve Stimulation – The Clinical Data

**Fig. 1:**
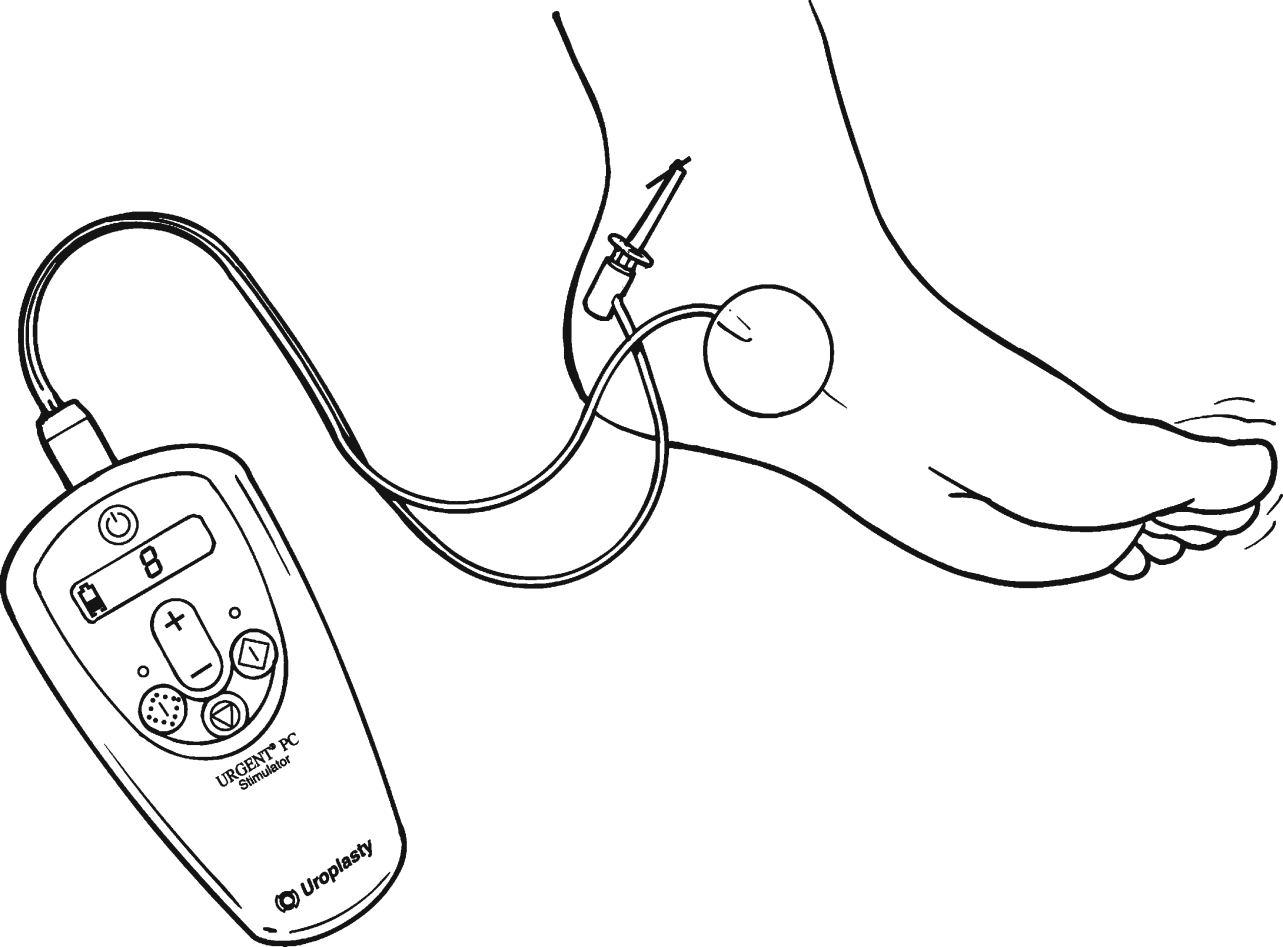
Percutaneous tibial nerve stimulation in the outpatient setting. *Reprinted with* *permission from* *Peters et* *al* [5••]

Percutaneous tibial nerve stimulation therapy is provided in the outpatient clinic setting. A 34-gauge needle electrode is inserted approximately 5 cm cephalad to the medial malleolus and posterior to the tibia (Fig. 1) with a surface electrode on the arch of the foot; percutaneous nerve stimulation at a current level of 0.5–9 mA at 20 Hz is performed initially for 30 minutes weekly for 12 weeks, followed by occasional treatments as needed based on patient symptoms [4••, 5••, 6••].

The basic science supporting PTNS has been well described in the published literature. In 1966, McPherson first demonstrated in a cat model that stimulation of the cut ends of dorsal spinal roots or various peripheral nerves including the posterior tibial nerve, effectively inhibited bladder contractions [15]. This effect was hypothesized to be mediated by neural circuitry in the forebrain, as intercollicular decerebration or thoracic spinal cord transection abolished the effect [16]. In 1980, Sato and colleagues verified that electrical stimulation of afferent nerves to hind limb muscles, but not cutaneous afferents, inhibited reflex bladder activity in the anesthetized cat [17]. In 1983, McGuire and Morrissey used electrical stimulation of the hindquarter nerves to treat detrusor instability in spinal injured nonhuman primates [18], and then went on to demonstrate this effect in 16 humans [19]. These exploratory studies were followed by multiple case series reports, and then by randomized, controlled trials.

The posterior tibial nerve is a mixed sensory-motor nerve, containing axons passing through the L4–S3 spinal roots. The sacral roots also contain the peripheral nerves involved in the sensory and motor control of the bladder and pelvic floor, and are the same spinal tracts targeted by sacral neuromodulation. Electrical stimulation of these nerves inhibits bladder activity by stimulating large diameter somatic afferent fibers, which in turn evokes a central inhibition of the micturition reflex pathway in the spinal cord or the brain. Although it is likely that stimulation of the sacral roots, (SNS), stimulation of the pudendal nerve, and stimulation of the tibial nerve (PTNS) all affect central components of the neural circuits controlling the bladder, there may be significant differences. A recent study in a cat model by Tai et al. demonstrated that both 5 and 30 Hz stimulation of the posterior tibial nerve inhibits bladder activity; however, stimulation of the pudendal nerve inhibits bladder activity at 5 Hz but excites bladder activity at 20-30 Hz [20]. In addition, repeated short duration stimulation of the tibial nerve induces a persistent post-stimulation inhibitory effect, and increases bladder capacity [20]. Unlike the persistent inhibition seen with tibial nerve stimulation, it appears that any change induced by SNS persists only while the stimulator is turned on, returning to baseline in the absence of chronic stimulation [21].

While the persistent inhibitory effects of tibial nerve stimulation and the transient clinical effect of SNS are dependent on somatic afferent modulation of spinal cord reflexes and brain networks, the synaptic mechanisms are unclear. The neural switching circuit for controlling bladder capacity is located in the pontine micturition center (PMC), and Tai et al. conclude that it is likely that the increased bladder capacity results from direct modulation of the PMC gating circuit or suppression of afferent input to that circuit [20]. The stimulation locus (peripheral nerve vs. spinal nerve root) and protocol (weekly and intermittent vs. chronic and continuous) are quite different for PTNS and SNS, and may utilize different spinal cord transmission routes and thus induce central modulation in different ways. Further study is required to understand the mechanisms of action and determine the physiological reasons for the differences seen in stimulation requirement and durability of response.

## Comparative Safety and Effectiveness of Current Therapies for OAB

Most efficacy studies of OAB therapies present objective measures, such as reduction in voids per day, nocturia, and episodes of urge incontinence, as well as subjective measures of the patient’s perceived improvement in health status. Although the ICS definition does not include urodynamic criteria, some studies provide these data (volume of void, maximum volume to trigger contraction) [7••, 22, 23]. Subjective measures (reduction in perceived symptoms and improvement in quality of life) are widely used, and may represent the most valuable outcome measure [24, 25]. The placebo or sham effect is particularly strong in OAB treatment. A recent review of the placebo effect in such trials reported that placebo pharmacologic treatment of urinary tract symptoms yielded reductions in incontinence episodes of 32-65 %, highlighting the need for an active comparative arm in any OAB treatment study [24]. The SUmiT Trial compared PTNS to a validated sham procedure and demonstrated PTNS superiority to sham for both objective voiding parameters and subjective patient assessments [5••]. In contrast, SNS studies do not include true placebo or sham arms, thus treatment effect size cannot be assessed.

## Outcome Measures––Objective Results

### Percutaneous Tibial Nerve Stimulation

There are over 30 studies of PTNS in the published literature; the earliest are case series or single arm efficacy studies [3, 7••, 23], three are randomized, controlled trials (RCT) [2, 5••, 6••], and two are long-term follow-up studies of patients who were responders in the OrBIT and SUmiT Trials [4••, 26••]. Over half of the patients receiving PTNS therapy in the SUmiT trial, a randomized, double-blinded, sham controlled study, reported moderate or marked improvement in bladder symptoms (54.5 % PTNS patients vs. 20.9 % sham, p < 0.001) [5••]. In addition, PTNS reduced the number of voids per day from 12.3 at baseline to 9.8 at 12 weeks, a mean reduction of -2.4 vs. a reduction of -1.5 in the sham group (p < 0.001). Urge incontinence episodes per day decreased from 3/day at baseline to 0.3/day at 12 weeks vs. 1.8/day at baseline to 1.0/day for sham (p < 0.001) [5••]. In a randomized, controlled study, Finazzi-Agro et al. report that PTNS significantly increased voided volume compared to sham treatment (150 mL to 186 mL in the PTNS treatment group vs. 146 mL to 150 mL in the sham group, p <0.001) [2]. In a urodynamic study, Klingler et al. report that PTNS increased mean total bladder capacity from 197 mL at baseline (range 35–349) to 252 ml (range 78–384 mL, p < 0.01) after 12 weeks of therapy [22]. When PTNS was compared against tolterodine extended release in the OrBIT study, both therapies demonstrated statistically significant improvements in incontinence episodes, voids per day, and nocturia [6••]. Although the gains in voided volume appear to be equivalently modest for both PTNS and for antimuscarinics, these gains are associated with significant improvements in subjective measures (see below), underscoring that these changes are clinically meaningful. The demonstrated increase in voided volume potentially represents an additional hour of bladder capacity.

### Antimuscarinic Therapy

The results for PTNS are comparable to those found in Chapple’s recent systematic review and meta-analysis of antimuscarinic drugs [27•]. A recent meta-analysis concluded that the significant improvement in OAB symptoms is comparable between PTNS and antimuscarinics, but that PTNS has a better adverse event profile [28]. Antimuscarinic drugs effect a reduction in incontinence episodes moderately better than that seen with placebo, but with bothersome adverse events for many patients. The relative improvement in objective measures from baseline above and beyond the placebo effect was modest but consistent across drugs and doses. A recent AHRQ Comparative Effectiveness review concluded that the benefits seen with antimuscarinics are small with no clear evidence for differential efficacy [29]. Urodynamic results are similar to those obtained with PTNS, in that pharmacological treatments typically increase voided volume by 13-39 mL [27•].

### Sacral Neuromodulation

SNS studies typically only include patients who have experienced a positive result with test stimulation, and thus do not include “non-responders” in intent-to-treat outcome rates [21]. Virtually none of the studies include a true sham group, as subjects can appreciate when the SNS electrode is stimulated. A recent Cochran review of the randomized SNS studies concluded that none were of adequate quality due to lack of sham groups and/or blinding [21]. In a recent review by Cameron of Medicare medical claims data, only 45.8 % of the percutaneous test stimulation patients went on to permanent implantation, and only 35.4 % of those with two-stage test stimulation underwent subsequent implantation [30]. Despite these limitations, the reported results indicate that 80–90 % of patients who received a permanent implant experienced a greater than 50 % improvement in their objective symptoms, with a significant decrease in incontinent episodes per day [21, 31]. Kessler, in a meta-analysis of the same studies reported that the pooled success rate for the test period was 68 %, and 92 % for permanent SNS [32].

## Outcome Measures––Subjective Results (Quality of Life)

Although subjective measures of health-related quality of life (HRQL) are often viewed as less reliable than objective ones, Van Leeuwen and colleagues note that OAB symptoms primarily affect quality of life (QOL), and that “assessing QOL and patient satisfaction should constitute an integral part of treatment efficacy assessment [24].” Virtually all trials of therapy for overactive bladder include at least one subjective measure of symptom reduction or HRQL in addition to the objective measures. Direct comparison of HRQL measures between studies is limited by the wide variety of instruments used; however all of these measures are validated, as are those used in PTNS studies [27•, 33, 34•]. A clear relationship between subjective and objective outcome measures has been demonstrated, such that therapies that show objective improvement in voids or incontinence episodes per day also find significant improvement in HRQL measures [5••, 6••, 34•]. Often the benefit seen in the HRQL measure is more positive than that seen with the objective measures. This may be related to a lessening of the distressing urge symptoms, thus patients are more comfortable throughout the day, even though they may void only 1–2 times less a day. A recent meta-analysis of the impact of antimuscarinic drugs on HRQL measures found that antimuscarinics improve several areas of HRQL, that these improvements are likely to be clinically meaningful, and that there were not significant differences between antimuscarinic drugs in degree of impact [35].

Similarly, the three randomized studies of PTNS included HRQL measures, and found that symptom severity score was significantly reduced when compared to sham treatment [5••], and mean quality of life was improved [2]. In the OrBIT trial comparing PTNS to tolterodine ER (4 mg/day), 79.5 % of women considered themselves cured or improved with PTNS versus only 60.5 % of tolterodine patients [6••]. In addition, subjective improvement was greater with PTNS than tolterodine for overall HRQL, and for each of four subscales (coping, concern, sleep and social) [6••].

The HRQL changes associated with SNS are inconsistent. In their review of the SNS studies that report quality of life changes with SNS, Herbison and Arnold note that it is difficult to know how many separate studies of SNS exist, as at least three appear to be sub-group analyses of the same study [21]. In one report, the effect on general health status as measured by the SF-36 was not significant, while in another it was highly significant. Given these inconsistencies between reports from the same study, Herbison and Arnold conclude that the effect of SNS on quality of life is still unclear [21].

## Long-Term Durability

In a 12-month study of patients who were responders to PTNS in the OrBit trial, on-going therapy with PTNS at lengthening intermittent intervals (average interval of 24.7 days) resulted in a sustained therapeutic effect in daily voids, urge incontinence episodes, and nocturia [4••]. A similar study conducted in patients who were responders in the SUmiT trial demonstrated sustained safety and efficacy of PTNS through 24 months, with an average of 1.3 treatments per month [26••].

Few data exist regarding the long-term durability of any pharmacological treatment of OAB; in the Chapple review of antimuscarinic trial reports, only two trials had durations greater than 12 weeks (16 and 52 weeks), providing few data about long term durability of drug therapy [27•]. Although the drug effect may be sustained, Gopal et al. report that 77.2 % of patients discontinue use within the first year with a mean of only 4.8 months of treatment [36•]. As with drug therapy, SNS must be continued for the duration of the patient’s life in order to be effective, and re-operation to change the stimulator battery is required every 3–7 years. Two long-term studies of SNS report a decrease of 31–45 % in mean and median voids per day at six months and two years, with a slight decrease in efficacy at five years (23 % decrease in symptoms from baseline) [21].

## Adverse Effects

Adverse events associated with PTNS are reported as mild, transient and relatively uncommon at 1–2 %, including bruising or bleeding at needle site, tingling and mild pain [5••, 6••]. The long-term durability studies reported similar mild and transient effects [4••, 26••]. Reports of adverse effects of antimuscarinics range from 9.7–63 %, with the most common adverse effects being constipation, dry mouth, impaired urination, and urinary tract infection [27•]. Serious adverse events were rare, but virtually all studies show a significantly higher rate of any adverse event with antimuscarinic therapy versus placebo [37]. The increased rate of adverse events with drug therapy may explain the high discontinuance within the first year of use [35].

Adverse events in SNS patients include pain at the implant site (15.3 %), new pain (9 %), suspected lead migration (8.4 %), infection (6.1 %), transient sensation of electric shock (5.5 %) and pain at lead site (5.4 %) [21], with 33–67 % of patients requiring surgical revision of the implant or leads within five years (includes battery changes) [37].

## Relative Cost of Therapy

As presented in the American Urological Association (AUA) Guidelines for treatment of OAB [1••], the syndrome of OAB represents a continuum of symptoms, with various therapies instituted along the treatment algorithm, and with varying costs. Behavioral therapies are noninvasive and inexpensive, but not free, as they require dedicated staff for training and follow-up. This conservative therapy can and should be offered to patients at any point in their care. Treatment with generic tolterodine is approximately $1200 per year, while the newer, branded medications may cost more than $200 per month [38]. The overall medical costs for OAB patients receiving antimuscarinic therapy, including office and ER visits, teaching, and laboratory costs, is estimated to be approximately $2,000 more than behavioral therapy alone [37].

A cost analysis of PTNS has not been published. Using the recently published 2012 Medicare Physician Fee Schedule with 3.80 total RVUs (relative value units) assigned to CPT Code 64566 and the Medicare national average physician in-office reimbursement of $134 per treatment, the cost of the first year of PTNS treatment would be approximately $3,500. This includes the initial 12 treatments, followed by an average of 12 treatments over the next 9 months and a total of five office visit charges throughout the therapy. Subsequent costs taper off with the diminishing requirements of ongoing therapy. Patients who elect to discontinue therapy can do so without additional surgical intervention or costs. SNS therapy is more expensive when discontinued, as corrective surgery to remove the electrodes and neurostimulator is required.

A recent cost analysis of interventions for antimuscarinic refractory patients reported SNS to be the most expensive, at a base rate of $26,269 for 3 years therapy (initial implantation plus revisions and management of adverse events) [39]. Augmentation cystoplasty, a more invasive surgical procedure, was estimated to cost $14,337 over 3 years, with botulinum toxin injection (not currently an FDA cleared therapy for idiopathic OAB) costing $7,651 over 3 years [39].

## OAB Algorithm

**Fig. 2:**
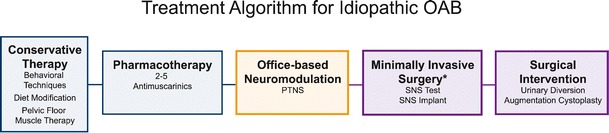
OAB treatment algorithm, progressing from the least expensive and noninvasive therapy of lifestyle and behavioral changes, through drug therapy, intermittent PTNS, surgically implanted SNS, and finally surgical interventions. (*Botulinum is not in the treatment algorithm as it is not currently an FDA approved therapy for idiopathic OAB.)

**Fig. 3:**
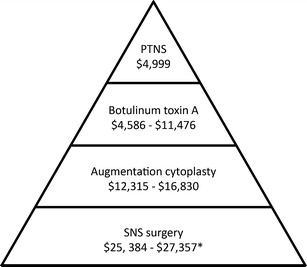
Relative costs of therapeutic options for overactive bladder

Patients with overactive bladder present with an entire spectrum of symptoms, from mild to severe frequency and urgency with or without mild to debilitating urge incontinence. As outlined by the AUA Guidelines, the OAB algorithm of care must include treatment options based upon individual presentation and expectations of treatment outcomes [1••]. As shown in Fig. 2, these treatment options move through increasingly invasive and expensive levels (Fig. 3), from lifestyle and behavioral changes through pharmacotherapy, office-based neuromodulation with PTNS, surgical implantation (SNS) and other surgical options. Although the AUA OAB algorithm suggests both PTNS and SNS can be offered as equivalent third tier options, our algorithm suggests that PTNS should be considered as a treatment option prior to SNS, due to the fact that PTNS is less invasive, has fewer adverse events, and a lower cost than SNS.

Approximately 40 % of the 34 million adult patients with OAB actively seek treatment. Lifestyle and behavioral interventions can influence voiding parameters at minimal expense to the patient and healthcare system, and should be offered at the beginning of care. Patients who continue to have troubling symptoms should be offered antimuscarinic drugs, which have demonstrated significant objective and subjective benefit, albeit modest [27•]. Persistence with anticholinergic drugs appears to be low, however, with poor adherence to medication for those who begin drug therapy; this patient population would benefit from additional effective therapies.

As described earlier, there are two types of neuromodulation that have been proven to be clinically effective. While there are no head-to-head RCTs that compare PTNS and SNS, it seems reasonable that patients should first be offered a treatment regimen of PTNS, as it is office-based, less invasive, and less expensive than SNS. The evidence reviewed above demonstrates that PTNS can effectively reduce OAB symptoms in the majority of patients within weeks of beginning therapy and that this benefit is sustained over time in responders. A recent study also showed that PTNS can be effectively added to drug therapy, gaining additional benefit when compared to drug alone, and that PTNS may permit a lower dose of drug for equivalent relief [40]. If peripheral neuromodulation proves to be ineffective, patients can subsequently be treated with the more invasive, permanent and costly SNS, with augmentation cystoplasty as the final most invasive option.

## Conclusions

The impact of OAB is profound in both personal and economic terms. The existence of more than 30 FDA-cleared therapies highlights the need for evidence based treatment options and an algorithm for the logical and efficient institution of therapy. Although antimuscarinic therapy is effective, pharmacological therapy will often not meet patient expectations. While SNS has been shown to be efficacious, the relative financial cost and the need for surgical implantation and surgical revision should be considered. PTNS provides an option for patients who are refractory to anticholinergic therapy; it is less invasive and less costly than SNS, and should be positioned early in the treatment algorithm of care for OAB.
